# A systematic review of GWAS identified SNPs associated with outcomes of medications for opioid use disorder

**DOI:** 10.1186/s13722-021-00278-y

**Published:** 2021-11-27

**Authors:** Caroul Chawar, Alannah Hillmer, Stephanie Sanger, Alessia D’Elia, Balpreet Panesar, Lucy Guan, Dave Xiaofei Xie, Nandini Bansal, Aamna Abdullah, Flavio Kapczinski, Guillaume Pare, Lehana Thabane, Zainab Samaan

**Affiliations:** 1 Neuroscience Graduate Program, McMaster University, Hamilton, ON Canada; 2 Department of Psychiatry and Behavioural Neurosciences, St. Joseph’s Healthcare Hamilton, 100 West 5th St., Hamilton, ON L8N3K7 Canada; 3 Health Sciences Library, McMaster University, Hamilton, ON Canada; 4 Health Sciences Program, McMaster University, Hamilton, ON Canada; 5 Population Health Research Institute, Hamilton, ON Canada; 6 Department of Health Research Method, Evidence, and Impact, McMaster University, Hamilton, ON Canada; 7 Father Sean O’Sullivan Research Centre, St. Joseph’s Healthcare Hamilton, Hamilton, ON Canada

**Keywords:** Opioid, Pharmacogenetic, MOUD, Methadone, GWAS, Systematic review

## Abstract

**Background:**

Patients with opioid use disorder (OUD) display an interindividual variability in their response to medications for opioid use disorder (MOUD). A genetic basis may explain the variability in this response. However, no consensus has been reached regarding which genetic variants significantly contribute to MOUD outcomes.

**Objectives:**

This systematic review aims to summarize genome-wide significant findings on MOUD outcomes and critically appraise the quality of the studies involved.

**Methods:**

Databases searched from inception until August 21st, 2020 include: MEDLINE, Web of Science, EMBASE, CINAHL and Pre-CINAHL, GWAS Catalog and GWAS Central. The included studies had to be GWASs that assessed MOUD in an OUD population. All studies were screened in duplicate. The quality of the included studies was scored and assessed using the Q-Genie tool. Quantitative analysis, as planned in the protocol, was not feasible, so the studies were analyzed qualitatively.

**Results:**

Our search identified 7292 studies. Five studies meeting the eligibility criteria were included. However, only three studies reported results that met our significance threshold of p ≤ 1.0 × 10^–7^. In total, 43 genetic variants were identified. Variants corresponding to *CNIH3* were reported to be associated with daily heroin injection in Europeans, *OPRM1*, *TRIB2*, and *ZNF146* with methadone dose in African Americans, *EYS* with methadone dose in Europeans, and *SPON1* and intergenic regions in chromosomes 9 and 3 with plasma concentrations of S-methadone, R-methadone, and R-EDDP, respectively, in Han Chinese.

**Limitations:**

The limitations of this study include not being able to synthesize the data in a quantitative way and a conservative eligibility and data collection model.

**Conclusion:**

The results from this systematic review will aid in highlighting significant genetic variants that can be replicated in future OUD pharmacogenetics research to ascertain their role in patient-specific MOUD outcomes.

*Systematic review registration number* CRD42020169121.

**Supplementary Information:**

The online version contains supplementary material available at 10.1186/s13722-021-00278-y.

## Background

### Rationale

Opioid use has been on the rise over the past decade, causing the United States and Canada, amongst other countries, to declare an opioid crisis and epidemic [1, 2]. In a 2019 report, the United Nations estimated about 53 million past-year users of opioids for 2017 worldwide [3]. That same year, 110,000 deaths were attributed to opioid use [3].

Treatments for opioid use disorder (OUD) have become more available and accessible under the term medication-assisted treatments or medications for opioid use disorder (MOUD). MOUD include the controlled administration of an opioid agonist or antagonist along with behavioural therapy or counselling with the objective of full recovery from opioid use [4]. Pharmacological agents of MOUD include the commonly used methadone, buprenorphine, buprenorphine/naloxone combination, naltrexone, heroin-assisted treatment, and sustained release morphine.

MOUD decreases the risk of overdose and mortality in individuals with OUD [5, 6]. A recent systematic review has reported the pooled overdose crude mortality rates for individuals being treated with MOUD compared to after the cessation of MOUD and during untreated periods being 0.24, 0.68, and 2.43, respectively [5]. Another review summarizing MOUD effectiveness in randomized controlled trials reported that the administration of MOUD at least doubles the rates of opioid abstinence when compared to placebo medications or no medications [6].

MOUD initiation and termination are important stages in determining patient health outcomes. As mentioned earlier, mortality risks tend to spike shortly after MOUD cessation [5]. Additionally, induction of methadone has shown an increased risk of overdose in multiple studies [7, 8]. Methadone dosing can affect electrocardiographic QTc interval prolongation, inducing respiratory depression amongst patients and increasing the risk for overdose mortality [9]. This is indicative that perhaps dosing of MOUD and its metabolism in patients are important factors in determining patient outcomes.

Given the individual basis of the treatment administration, a genetic predisposition to MOUD responses may be involved. OUD is a complex polygenic disorder with not one genetic variant attributing to a large risk or effect. Genetic association studies researching genetic variants or single-nucleotide polymorphisms (SNPs) associated with OUD or its treatment outcomes require large sample sizes to generate enough power to identify such variants [10].

Currently, the most common SNPs associated with MOUD outcomes correspond to *OPRM1*, *OPRD1*, *ABCB1*, and *CYP2B6* genes [11, 12]. *OPRM1*, *ABCB1*, and *CYP2B6* variants have been associated with altered methadone doses [12]. *ABCB1* along with *CYP2B6* variants have also been linked to variable methadone plasma concentrations. Other studies showed variants in *OPRD1* to be associated with opioid-positive urine screens and therapeutic responses in patients administered methadone versus buprenorphine [11, 12].

Though there seem to be numerous studies assessing the pharmacogenetics of MOUD, many of which are candidate gene studies with small samples sizes. To produce replicable results and discover new significantly associated SNPs, robust genome-wide association studies (GWASs) need to be performed and assessed. This systematic review is the first to summarize the current literature, assess the quality of the findings, and report on the areas that need to be addressed within this field.

### Objectives

The aims of this systematic review are to highlight any significant GWAS genetic variants that are associated with MOUD outcomes in patients, including illicit opioid use as well as secondary outcomes such as MOUD plasma concentrations and doses [13].

The specific objectives are:

1. Summarize the genome-wide significant SNP outcome associations reported in the literature and highlight novel ones.
2. Critically examine and assess the quality of the findings extracted within the relevant studies using the Q-Genie tool.
3. Identify gaps within the literature that need to be addressed with respect to pharmacogenetic research of MOUD outcomes.

## Methods

This systematic review is reported in accordance with the Preferred Reporting Items for Systematic Reviews and Meta-Analyses (PRISMA) guidelines [14]. A supplementary PRISMA checklist is in Additional File 1. Since the focus of this review is on GWASs, it does not conform with the Human Genome Epidemiology Network (HuGENet) guideline expectations of reporting on candidate gene study findings [15]. However, the HuGENet guideline is used to supplement the PRISMA guidelines, to provide a more informed review, upholding a standard of reporting specific to genetic association studies.

### Protocol and registration

This systematic review has been registered with the International Prospective Register of Systematic Reviews (PROSPERO) [16]; registration ID CRD42020169121. A systematic review protocol has been published in *Systematic Reviews* [13]. The detailed methods of this systematic review are specified and documented in the registration and protocol.

### Eligibility criteria and search strategy

The eligibility for inclusion in this systematic review is three-fold. The study design of included studies is limited to GWASs specific to genetic variants of interest reported as SNPs. The included studies have to look at an OUD population. Lastly, included studies have to investigate a MOUD, such as methadone, buprenorphine/naloxone, buprenorphine, naltrexone, or heroin-assisted treatment. Studies are not restricted by language, patient demographics, or MOUD administration setting.

A search strategy was developed with help from a Health Sciences Librarian (SS). Table 1 outlines the databases searched and the search terms used. All databases were searched from inception to August 21st, 2020. Handsearching was used to identify relevant studies that were not detected by the search strategy, such as those assessing sustained-release morphine as a treatment.

**Table 1:** Search strategy

Medline (Ovid)
1. Genome-Wide Association Study/
2. Genotyping Techniques/
3. Genome, Human/
4. Genetic Variation/
5. Genetics/ or exp human genetics/
6. (Human* adj2 (genotyp* or genome* or genetic*)).ti,ab,kw,kf
7. (GWS or GWAS or GWA).mp
8. Genome wide.ti,ab,kw,kf
9. 1 or 2 or 3 or 4 or 5 or 6 or 7 or 8
10. Exp Opioid-Related Disorders/
11. ((Opiate* or opioid* or heroin* or codeine* or dilaudid* or fentanyl* or narcotic* or drug* or substance*) adj2 (overdose* or use* or using or misuse* or abus* or dependence* or addict*)).ti,ab,kw,kf
12. Opiate Substitution Treatment/
13. ((Opiate* or opioid*) adj2 (treatment* or therap*)).ti,ab,kw,kf
14. Exp buprenorphine/ or exp naloxone/
15. Exp Methadone/
16. (Suboxone or methadone or buprenorphine or naloxone).ti,ab,kw,kf
17. 10 or 11 or 12 or 13 or 14 or 15 or 16
18. 9 and 17
19. *Limit 18 to humans*
Web of science—All databases
1. TS = (genome-wide association study or genome-wide association or GWAS or GWA or genome wide or genome)
2. T S = ((opiate* or opioid* or heroin* or fentanyl* or narcotic* or drug* or substance*) NEAR/2 (overdose* or use* or using or misus* or abus* or dependence* or addict*))
3. TS = ((treatment* or therap*) NEAR/2 (opiate* or opioid* or heroin* or fentanyl* or narcotic* or drug* or substance*)
4. TS = (methadone or buprenorphine or naloxone or naltrexone or heroin-assisted or suboxone)
5. #3 or #4
6. #1 and #2 and #
EMBASE (Ovid)
1. Genome-Wide Association Study/
2. Genotyping Techniques/
3. Genome, Human/
4. Genetic Variation/
5. Genetics/ or exp human genetics/
6. (Human* adj2 (genotyp* or genome* or genetic*)).ti,ab,kw
7. (GWS or GWAS or GWA).mp
8. Genome wide.ti,ab,kw
9. 1 or 2 or 3 or 4 or 5 or 6 or 7 or 8
10. Exp Opioid-Related Disorders/
11. ((Opiate* or opioid* or heroin* or codeine* or dilaudid* or fentanyl* or narcotic* or drug* or substance*) adj2 (overdose* or use* or using or misuse* or abus* or dependence* or addict*)).ti,ab,kw
12. Opiate Substitution Treatment/
13. ((Opiate* or opioid*) adj2 (treatment* or therap*)).ti,ab,kw
14. Exp buprenorphine/ or exp naloxone/
15. Exp Methadone/
16. (Suboxone or methadone or buprenorphine or naloxone).ti,ab,kw
17. 10 or 11 or 12 or 13 or 14 or 15 or 16
18. 9 and 17
19. *Limit 18 to human*
CINAHL and Pre-CINAHL
1. Genome-wide association study or genome-wide association or GWAS or GWA or genome wide or genome
2. Opiate* or opioid* or heroin* or fentanyl* or narcotic* or drug* or substance*
3. Overdose* or use* or using or misus* or abus* or dependence* or addict*
4. S2 and S3
5. Treatment* or therap*
6. S5 and S2
7. Methadone or buprenorphine or naloxone or naltrexone or heroin-assisted or suboxone
8. S6 or S7
9. S1 and S4 and S8
10 .*Limit to Human*
GWAS Catalog—publications
-Methadone
-Opioid
-Heroin
-Drug abuse
GWAS Central—studies list
-Methadone
-Heroin
-Opioid
-Opiate
-Addiction
-Drug abuse
-Opioid dependence
-Opioid addiction
-Fentanyl
NIH Database of genotypes and phenotypes
-Search (opioid)
-Search (heroin)

### Data collection and outcomes

Title and abstract screening, full-text screening, and data extraction of studies were all completed in duplicate via Covidence [17]. The voting of reviewers remained blinded and conflict resolution for the screening stages was performed by a senior reviewer (AH or CC), keeping the process unbiased. Authors of full text articles that were not found or unavailable were contacted regarding the provision of the full text so the study can better assess them for inclusion in this systematic review. The data extraction form was pilot tested in duplicate prior to proceeding with data collection.

Data extracted include study information, baseline participant characteristics, relevant and significant measured outcomes, statistical measures, and reported study limitations and conflicts. For the purposes of this review, the significance threshold of SNP outcome associations extracted is p ≤ 1 × 10^–7^, as some GWAS results with this significance level have been shown to be replicable within the literature [18].

The outcomes of interest in this review pertain to genetic variants significantly associated with MOUD outcomes observed in OUD patients. The primary MOUD outcome considered is illicit opioid use or abstinence during or following MOUD. The secondary MOUD outcomes include time to relapse, treatment retention, opioid overdose, non-opioid substance use, comorbid psychiatric disorders, drug-related risk-taking behaviours, MOUD and metabolite plasma concentration, MOUD dose, and mortality.

### Quality assessment and data analysis

Quality assessment of each included study is done using the Quality of Genetic Association Studies (Q-Genie) tool [Version 1.1], assessing the study validity, reliability, and risk of bias [19]. Quality assessments are completed in duplicate, and conflicts regarding the scoring are resolved by the reviewers.

A heterogeneity test and random effects meta-analysis through pooled odds ratios or calculated mean differences, respective of the measure of association, were planned to quantitatively assess the data, as outlined in the protocol. However, these measures were not appropriate as data extracted from each study was unique and could not be synthesized.

For the aforementioned reasons, subgroup meta-analyses and risk of bias assessments across studies also could not be completed.

## Results

### Study selection

**Fig. 1:**
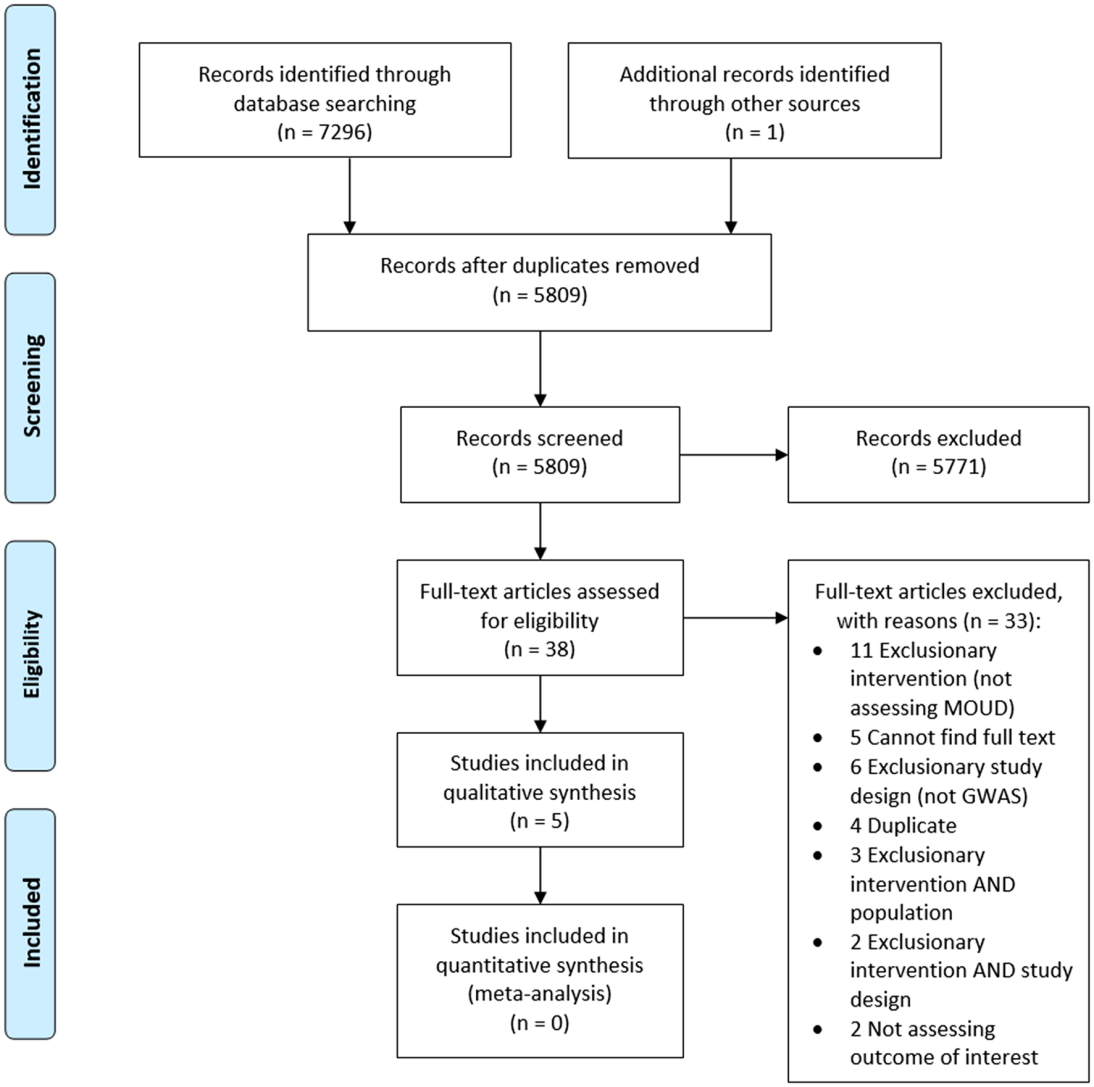
PRISMA flow diagram of study inclusion

A total of 5 studies were eligible for inclusion in this systematic review [20–24]. The search strategy along with handsearching techniques identified 7292 studies, with 5809 advancing to the title and abstract screening after the removal of duplicates by both the Zotero reference manager and Covidence [17, 25]. Following title and abstract screening, 38 studies were deemed relevant for full-text screening, and 5771 studies were excluded due to not being GWASs, not assessing an OUD population, and/or not assessing a MOUD. Of the 38 full-text studies assessed for eligibility, 5 GWASs (3 prospective, 1 cross-sectional, and 1 case–control) underwent data extraction and qualitative assessment. See flow diagram in Fig. 1.

### Study characteristics

Table 2 provides a summary of the included study characteristics. All five studies were published in English. Three were prospective studies, one case–control, and one cross-sectional. The sample size studied varied from a few hundred to thousands of participants, the smallest being 344 and largest 4049. All studies had a majority male study population, varying from 59.72% to 81.6% males. The mean age per studied population varied from 33.03 (5.45) to 45.6 (8.4). Ancestries of the participants included in these GWASs were European, African American, and/or Han Chinese, with Europeans constituting the largest sample. Two of the studies identified used the same sample population of Han Chinese individuals for their analyses, though performed different statistical measures [23, 24]. Three of the studies reported that participants were administered methadone as their MOUD [22–24], and two did not specify [20, 21]. The outcomes of interest that were associated with genetic variants were opioid cessation, daily heroin injection while on MOUD, methadone dose, and plasma concentrations of methadone and its metabolite EDDP. No study assessed relapse, treatment retention, opioid overdose, non-opioid substance use, psychiatric disorders, risk-taking behaviours, or mortality as outcomes associated with genetic variation.

**Table 2:** Summary of included studies

First author last name, year of publication	N cases/controls	% Male	Mean age (SD)	Ethnicity	Type of MOUD	Study design	Relevant outcomes measured
Cox, 2020 [20]	4049	63.45%	NA	African American = 1130, European = 2919	Opioid Substitution Treatment (unspecified)	Prospective	Opioid cessation - USA sample: defined as self-reported abstinence from illicit opioids for > 1 year (ceased) or < 6 months (not ceased) before the interview date - Australia sample: self-reported last use of an opioid was at least one year before the age at the interview (ceased) or the age of last use of an opioid was the same as the age at the interview (not ceased)
Nelson, 2016 [21]	1167 cases, 161 controls	60.1%	36.9 (8.4)	European	Methadone or Buprenorphine Opioid Replacement Therapy (cases)	Case–control	Continued opioid use (OD_E_ – self-reported daily heroin injection while on treatment)
Smith, 2017 [22]	1410	59.72%	AA: Males: 45.6 (8.4); Females: 43.0 (7.2) EA: Males: 37.2 (10.1); Females: 37.5 (9.8)	African American = 383, European American = 1027	Methadone	Prospective	Usual daily methadone dose (self-reported) (mg)
Wang, 2018 [23]	344	81.68%	38.17 (7.69)	Han Chinese (Taiwan)	Methadone	Cross-sectional	Methadone dose (obtained from participant medical record) (mg)
Yang, 2016 [24]	344	81.68%	Males: 39.31 (7.66); Females: 33.03 (5.45)	Han Chinese (Taiwan)	Methadone	Prospective	Plasma concentrations of methadone and its metabolite EDDP R- and S-enantiomers (measured using high-performance liquid chromatography) (ng/ml/mg/dose)

### Risk of bias within studies

The quality and validity of each study was assessed using the Q-Genie tool on a scale of 1 to 7 [19]. Studies with a control group and with overall scores of greater than or equal to 45, as well as studies with no control group with overall scores of greater than 40 were considered of good quality, per the tool classification guidelines. All but one study were assessed to be of good quality, while Nelson et al. was deemed to be of moderate quality [21]. It should be noted that the primary objectives of Nelson et al.’s study might not have been to assess an MOUD outcome per se, but rather opioid dependence end points amongst opioid-dependent daily injectors (cases) versus nondaily injecting opioid misusers (controls). However, due to satisfying the eligibility criteria and analyzing an outcome of interest to us in only the cases, this study was included. Three of the included studies report insufficient sample sizes that might result in not detecting genome-wide significant SNPs [22–24]. The three studies also disclose conflicts of interest that are reported to not be interferent with the research conducted [20–22]. See Table 3 for a summary of the reported limitations and conflicts of interest, as well as the quality assessments.

**Table 3:** Quality assessments and reported study limitations and conflicts

First author last name, year	Reported conflicts of interest	Reported study limitations	Q-Genie Score	Quality assessment
Cox, 2020	H.R.K. is a member of the American Society of Clinical Psychopharmacology’s Alcohol Clinical Trials Initiative, which was supported for the last three years by AbbVie, Alkermes, Ethypharm, Indivior, Lilly, Lundbeck, Otsuka, Pfizer, Arbor, and Amygdala Neurosciences. Drs. Kranzler and Gelernter are named as inventors on PCT patent application #15/878,640 entitled: "Genotype-guided dosing of opioid agonists," filed January 24, 2018. The funders had no role in the design of the study; in the collection, analyses, or interpretation of data; in the writing of the manuscript; or in the decision to publish the results	•Used cross-sectional data to study a phenotype that would require long-term follow-up to define cessation more accurately •Used a slightly different definition for cessation in the CATS dataset than in the Yale-Penn dataset •The opioid cessation GWAS sample had limited power to detect genome-wide significant association signals	63	Good quality
Nelson, 2016	Although unrelated to the current study, Dr Kranzler has been a consultant or advisory board member for Alkermes, Lilly, Lundbeck, Pfizer and Roche. He is also a member of the American Society of Clinical Psychopharmacology's Alcohol Clinical Trials Initiative, which is supported by Lilly, Lundbeck, Abbott and Pfizer. The remaining authors declare no conflict of interest	•Small size of control group (OU_IP_) •A more detailed characterization of the opioid use in the OU_IP_ group was not obtained	45	Moderate quality
Smith, 2017	Dr. Kranzler reports being a consultant, continuing medical education (CME) speaker, or advisory board member for Alkermes, Indivior, Lundbeck, and Otsuka, and a member of the American Society of Clinical Psychopharmacology’s Alcohol Clinical Trials Initiative, which was supported in the last three years by AbbVie, Alkermes, Ethypharm, Indivior, Lilly, Lundbeck, Otsuka, Pfizer, and XenoPort	•Small sample size compared to mega-GWASs with pooled data •Daily methadone dose was self-reported	47	Good quality
Wang, 2018	None	•No statistically significant GWAS findings that pass the threshold p < 3.2 × 10^–6^ •Small sample size •Most subjects were male and 95% tested positive for HCV •Study was cross-sectional in design	44	Good quality
Yang, 2016	None	•Moderate sample size •Small replication sample—may not have detected significant associations (insufficient power)	53	Good quality

### Results of individual studies

Of the five studies included, only three reported outcomes that reached the threshold of significance set for this systematic review (Table 4) [21, 22, 24].

**Table 4:** Summary of SNP outcome associations

First author last name, year	Outcome	SNP ID	Chr: position	Alleles	Minor allele	Gene	MAF	N	Measure of association	Measure of association value	Measure of variability	Measure of variability value	p-value	Ethnicity
Cox, 2020 [20]	NA													
Nelson, 2016 [21]	Opioid dependence end point (daily heroin injection)	rs1436171	1:224881828	A/?	A	*CNIH3*	0.44	1167	OR	0.54	95% CI	0.42–0.68	6.26E-07	European
		rs1369846	1:224894095	C/?	C	*CNIH3*	0.38	1167	OR	0.52	95% CI	0.41–0.66	9.42E-08	European
		rs1436175	1:224908366	T/?	T	*CNIH3*	0.37	1167	OR	0.5	95% CI	0.39–0.64	2.72E-08	European
Smith, 2017 [22]	Methadone dose	rs73568641	6:154025139	C/T	C	*OPRM1*	0.1	383	β	0.6808	SE	0.1226	2.81E-08	African American
		rs7451325	6:154016517	C/T	C	*OPRM1*	0.1	383	β	0.6807	SE	0.1226	2.83E-08	African American
		rs111559266	6:153998560	A/G	A	*OPRM1*	0.1	383	β	0.6546	SE	0.1252	1.72E-07	African American
		rs76499485	6:154004364	A/G	A	*OPRM1*	0.1	383	β	0.6487	SE	0.1257	2.48E-07	African American
		rs7578347	2:13121168	T/C	T	*TRIB2*	0.43	383	β	0.3926	SE	0.0764	2.77E-07	African American
		rs7578329	2:13121135	T/C	T	*TRIB2*	0.43	383	β	0.3924	SE	0.0764	2.81E-07	African American
		rs13423393	2:13120763	T/C	T	*TRIB2*	0.43	383	β	0.3922	SE	0.0764	2.85E-07	African American
		rs6745283	2:13120700	A/T	A	*TRIB2*	0.43	383	β	0.3923	SE	0.0765	2.93E-07	African American
		rs73568677	6:154046471	T/C	T	*OPRM1*	0.09	383	β	0.6431	SE	0.1261	3.42E-07	African American
		rs116777827	6:154084534	T/C	T	*OPRM1*	0.11	383	β	0.5981	SE	0.1176	3.64E-07	African American
		rs4669899	2:13121465	T/C	T	*TRIB2*	0.41	383	β	0.3928	SE	0.0779	4.62E-07	African American
		rs4669900	2:13121525	T/C	T	*TRIB2*	0.41	383	β	0.3925	SE	0.078	4.87E-07	African American
		rs4669901	2:13121591	G/A	G	*TRIB2*	0.41	383	β	0.393	SE	0.0781	4.88E-07	African American
		rs13397286	2:13120841	A/G	A	*TRIB2*	0.42	383	β	0.3926	SE	0.0781	5.01E-07	African American
		rs12664381	6:154054500	T/C	T	*OPRM1*	0.11	383	β	0.5873	SE	0.1171	5.26E-07	African American
		rs12527630	6:154064934	G/A	G	*OPRM1*	0.11	383	β	0.5868	SE	0.1172	5.50E-07	African American
		rs73570652	6:154070563	T/C	T	*OPRM1*	0.11	383	β	0.5867	SE	0.1172	5.52E-07	African American
		rs12663416	6:154057383	T/C	T	*OPRM1*	0.11	383	β	0.5856	SE	0.117	5.54E-07	African American
		rs12104412	19:36731058	T/A	T	*ZNF146*	0.15	383	β	0.4939	SE	0.099	6.00E-07	African American
		rs57072980	2:13122014	T/C	T	*TRIB2*	0.43	383	β	0.3823	SE	0.0772	7.36E-07	African American
		rs9360217	6:67338593	G/T	G	*EYS*	0.22	1027	β	− 0.2613	SE	0.0525	6.55E-07	European American
		rs9345875	6:67370087	G/T	G	*EYS*	0.21	1027	β	− 0.2602	SE	0.0524	6.95E-07	European American
		rs9342570	6:67368858	A/T	A	*EYS*	0.21	1027	β	− 0.2589	SE	0.0523	7.53E-07	European American
		rs9345867	6:67359694	C/T	C	*EYS*	0.21	1027	β	− 0.258	SE	0.0522	7.83E-07	European American
		rs2045196	6:67339443	G/C	G	*EYS*	0.21	1027	β	− 0.265	SE	0.0537	8.15E-07	European American
		rs1026388	6:67348220	A/C	A	*EYS*	0.21	1027	β	− 0.2576	SE	0.0523	8.55E-07	European American
		rs4142573	6:67388037	T/C	T	*EYS*	0.21	1027	β	− 0.2561	SE	0.052	8.57E-07	European American
		rs9363624	6:67387453	C/T	C	*EYS*	0.21	1027	β	− 0.2561	SE	0.052	8.57E-07	European American
		rs9354462	6:67383719	T/C	T	*EYS*	0.21	1027	β	− 0.2565	SE	0.0521	8.64E-07	European American
		rs9351587	6:67400119	T/C	T	*EYS*	0.21	1027	β	− 0.256	SE	0.052	8.65E-07	European American
		rs4710324	6:67352212	T/C	T	*EYS*	0.21	1027	β	− 0.2574	SE	0.0523	8.72E-07	European American
		rs9342572	6:67386966	T/C	T	*EYS*	0.21	1027	β	− 0.2574	SE	0.0523	8.72E-07	European American
		rs4710621	6:67389232	G/A	G	*EYS*	0.21	1027	β	− 0.2559	SE	0.052	8.74E-07	European American
		rs2124198	6:67366749	C/T	C	*EYS*	0.21	1027	β	− 0.2567	SE	0.0522	8.89E-07	European American
		rs9345880	6:67391212	C/T	C	*EYS*	0.21	1027	β	− 0.2556	SE	0.052	9.00E-07	European American
		rs9360224	6:67397651	T/C	T	*EYS*	0.21	1027	β	− 0.2553	SE	0.052	9.26E-07	European American
		rs2124199	6:67391889	A/T	A	*EYS*	0.21	1027	β	− 0.2539	SE	0.0518	9.65E-07	European American
Wang, 2018 [23]	NA													
Yang, 2016 [24]	Plasma concentration of R-methadone	rs17180299	9:NA	A/G	G	intergenic	0.09	344	β	NA	NA	NA	2.24E-08	Han Chinese
	Plasma concentration of S-methadone	AX-16534452	11:NA	NA	NA	*SPON1*	NA	344	β	NA	NA	NA	4.83E-07	Han Chinese
	Plasma concentration of R-EDDP	rs1448332	3:NA	NA	NA	intergenic	NA	344	β	NA	NA	NA	8.18E-07	Han Chinese

Chr is the chromosome number, and position refers to the SNP position on the respective chromosome. MAF is the reported minor allele frequency, N is the sample size of the analyzed population, and Outcome is the phenotype associated with the SNP. β refers to the beta coefficient, OR the odds ratio, SE the standard error, and 95% CI to the 95% confidence intervals reported. NA was used to designate missing information

Nelson et al. identified three SNPs associated with opioid dependence end point in the gene *CNIH3* (chromosome 1). The participants were daily heroin-injecting patients on methadone or buprenorphine of European ethnicity. The three SNPs reported are in moderate to high linkage disequilibrium, with the odds of the risk alleles being found in the daily heroin injecting group approximately 50% lower than in the control group [21].

Smith et al. identified thirty-seven SNPs associated with methadone dose in varying genes across methadone-treated African American and European American populations. Amongst participants of African American ethnicity, the SNPs correlated to the following genes: *OPRM1* (chromosome 6), *TRIB2* (chromosome 2), and *ZNF146* (chromosome 19). On the other hand, the SNPs identified in European Americans correlated to only one gene, *EYS* (chromosome 6). The leading SNP nearest to the *OPRM1* gene (rs73568641) was reported to be in mid to high linkage disequilibrium with neighbouring SNPs identified. Linkage disequilibrium amongst SNPs of other genes was not reported as they were not genome-wide significant. The presence of the risk alleles in the *OPRM1*, *TRIB2*, and *ZNF146* genes is observed to be associated with an increase in the usual daily methadone dose in African American patients. In contrast, the presence of the risk alleles in the *EYS* gene is observed to be associated with a decrease in the usual daily methadone dose in European Americans [22].

Lastly, Yang et al. identified three SNPs associated with methadone and EDDP plasma concentrations. The participants were methadone-administered patients in Taiwan of Han Chinese ancestry. One SNP was associated with plasma concentration of R-methadone, corresponding to an intergenic region (chromosome 9), one with plasma concentration of S-methadone, corresponding to the *SPON1* gene (chromosome 11), and the last one associated with plasma concentration of R-EDDP, corresponding to another intergenic region (chromosome 3). The measure and magnitude of association for these SNPs were not reported [24].

## Discussion

### Summary of evidence

Advances in pharmacogenetic research within OUD populations have been on the rise. Yet, no attempt has been made in quantitatively and qualitatively analyzing the literature and critiquing the quality of evidence reported by GWASs. This systematic review was able to summarize findings from GWASs with borderline genome-wide significance and the potential of being replicable in future studies. We have identified five studies that match our inclusion criteria, with three studies reporting significant results. SNPs associated with outcomes of daily heroin injection, methadone dose, and methadone and EDDP plasma concentration were found to be significant. SNPs corresponding to genetic regions of *CNIH3* were reported to be more prevalent in daily heroin injecting patients. SNPs corresponding to or near *OPRM1*, *TRIB2*, *ZNF146*, and *EYS* were associated with methadone dose levels, depending on ethnicity. SNPs in an intergenic region on chromosome 9, *SPON1*, and an intergenic region on chromosome 3 were associated with differing plasma concentration of R-methadone, S-methadone, and R-EDDP, respectively. The quality of research and reporting of each study was assessed with the Q-Genie tool and no study was deemed to be of poor quality. Varying sample sizes were however observed, with some being too small for what is considered acceptable for GWAS analysis. With sample sizes of thousands required to produce adequately powered results in GWASs [26], sample sizes from Yang et al. (n = 344) and the African American population of Smith et al. (n = 383) fell short.

One gene related to the SNPs identified has been reported previously within candidate gene studies and has an established biological relevance within the genetics and pharmacogenetics of OUD research. The *OPRM1* gene encodes the mu-opioid receptor, which binds endogenous and exogenous opioids [27]. Genetic variability in *OPRM1* has been reported to have biological effects on the mu-opioid receptor function contributing to complex disorders. An in-vitro study showed that the *OPRM1*-G118 variant reduces *OPRM1* mRNA and protein levels [28]. When studied in mice models, the equivalent point mutation *OPRM1*-G112 also resulted in decreased mu-opioid receptor mRNA and protein expression [29]. Findings showed that mice with the G112 allele had reduced morphine-induced antinociceptive responses [29]. Consistently, *OPRM1* has been reported to be highly influential in opioid dependency, and, by some findings, OUD treatment outcomes, such as methadone dose and plasma concentrations, in European patients [30]. Therefore, it is not a surprise for SNPs in this gene to be associated with methadone dose at a GWAS significance level. Though, Smith et al.’s results are interesting because they found an *OPRM1* association in patients of African American ethnicity but not of European ethnicity, as was expected. This incongruity calls for additional powered research in both ethnic populations to be conducted for a consensus.

Another gene identified has not been previously associated with OUD or MOUD outcomes in the literature but could be involved in biological pathways relevant to opioid use. The *CNIH3* gene encodes the protein cornichon homolog 3, which regulates AMPA receptor trafficking [27]. This gene has been identified in schizophrenia studies by NCBI’s Gene database [31]. Therefore, it is possible that *CNIH3* could be associated with the regulation of opioid use.

Most of the genes involving an identified SNP summarized in this systematic review do not seem to have been relevant to OUD or MOUD outcomes, nor could a biological relevance be identified for them. These genes include *TRIB2*, *ZNF146*, *EYS*, *SPON1*, as well as the intergenic regions for the SNPs located on chromosomes 3 and 9. The *TRIB2* gene encodes the tribbles homolog 2 protein that regulates MAP kinase proteins’ activation [27]. This gene is evident in many tissues, most prominently in the ovaries, spleen, and nymph node tissues [31]. It has also been reported in the NCBI Gene Database to be identified in studies researching schizophrenia, neuropsychiatric disorders, autism, and aging [31]. *ZNF146* encodes the zinc finger protein OZF, the primary function of which is to regulate DNA binding and transcription [27]. As such, it is present in a lot of tissues, including the brain, but is more prominent in the endometrium and thyroid [31]. In humans, *EYS* encodes the protein eyes shut homolog, which as deduced from the name, is involved in vision, more specifically, in maintaining the morphological integrity of photoreceptor cells through the possible involvement in channel regulations [27]. *EYS* is most prevalently expressed in fat and testis tissue [31], which shows no direct relation to methadone dose or metabolism as identified in Smith et al. Lastly, *SPON1* encodes spondin-1, which is a cell adhesion protein within the nervous system [27]. *SPON1* is mostly expressed in the gall bladder tissue [31], which does not provide a clear biological link to its function nor the outcome of methadone plasma concentration reported by Yang et al. [31]. Further research is required to make any conclusive statements concerning the biological relevance of SNPs in these genes to the observed MOUD outcomes.

In general, the results of this systematic review are able to inform future candidate gene studies and GWASs of key SNPs that require further research in larger cohorts as well as replications to solidify their associations to MOUD outcomes in indicated patients. The findings from such studies are able to inform the clinical and pharmacological response to patient doses and drug outcomes for administered MOUD.

### Limitations

Though rigorous, this systematic review has some limitations associated with the strict eligibility criteria predetermined in the protocol. It is important to note that in the process of including studies that were primary GWASs, GWAS meta-analyses were excluded. This could have affected the number, quality, and significance of the findings. An example is the exclusion of the GWAS meta-analysis findings from Nelson et al. that replicated original findings in a larger meta-analyzed sample, highlighting new SNPs that achieved significance (rs10799590, rs12130499, and rs298733) and SNPs that fell below our significance threshold in the process (rs1436175) [21]. However, since most GWAS meta-analyses reported associations using the same study populations and sample data, their inclusion would have made any reported findings redundant. Another limitation could be the exclusion of studies that reported genetic variance in the form of haplotypes. Though their inclusion might have made a meta-analysis possible, they did not satisfy the eligibility criteria of a SNP identified by a GWAS and would, therefore, not be very informative within the scope of our systematic review.

As stated previously, a meta-analysis was not feasible with the heterogeneity of the reported findings. This makes consensus more difficult to reach and the findings less generalizable, especially when considering differing ethnicities.

In addition, this systematic review was only able to highlight published GWAS associations. As a result, any findings that were not published due to inability to meet statistical thresholds might not have been included. Though efforts were made to include near genome-wide significant findings, the possible presence of publication bias should still be acknowledged.

## Conclusions

Through this systematic review, we were able to summarize GWAS significant findings in the field of OUD pharmacogenetics. We were able to inform the availability of data by highlighting what has been done within this research field, and what gap exists and needs to be addressed. Recommendations of further powered research are made, with close attention to the ethnicities of participating cohorts to test whether SNP outcome associations within one ethnicity hold competing levels of validity in another.

## Supplementary Information

**Additional file 1:** PRISMA 2009 Checklist.

## Data Availability

All data generated or analysed during this study are included in this published article.

## Abbreviations

OUD: Opioid use disorder
MOUD: Medications for opioid use disorder
SNP: Single nucleotide polymorphism
GWAS: Genome-wide association study
PRISMA: Preferred Reporting Items for Systematic Reviews and Meta-Analyses
HuGENet: Human Genome Epidemiology Network
PROSPERO: International Prospective Register of Systematic Reviews
Q-Genie: Quality of Genetic Association Studies
